# A review of anti-inflammatory, antioxidant, and immunomodulatory effects of *Allium cepa* and its main constituents

**DOI:** 10.1080/13880209.2021.1874028

**Published:** 2021-02-28

**Authors:** Narges Marefati, Vahideh Ghorani, Farzaneh Shakeri, Marzie Boskabady, Farzaneh Kianian, Ramin Rezaee, Mohammad Hosein Boskabady

**Affiliations:** a Applied Biomedical Research Center, Mashhad University of Medical Sciences, Mashhad, Iran; b Department of Physiology, School of Medicine, Mashhad University of Medical Sciences, Mashhad, Iran; c Natural Products and Medicinal Plants Research Center, North Khorasan University of Medical Sciences, Bojnurd, Iran; d Department of Physiology, School of Medicine, North Khorasan University of Medical Sciences, Bojnurd, Iran; e Dental Materials Research Center and Department of Pediatric Dentistry, School of Dentistry, Mashhad University of Medical Sciences, Mashhad, Iran; f Department of Pediatric Dentistry, School of Dentistry, Mashhad University of Medical Sciences, Mashhad, Iran; g Department of Physiology, School of Medicine, Tehran University of Medical Sciences, Tehran, Iran; h Clinical Research Unit, Faculty of Medicine, Mashhad University of Medical Sciences, Mashhad, Iran

**Keywords:** Onion, flavonoids, phenolic, inflammation, oxidative stress, quercetin

## Abstract

**Context:**

*Allium cepa* L. (Liliaceae), known as onion, is consumed throughout the world. Onion and its derivatives including saponins, aglycones, quercetin, cepaenes, flavonoids, organosulfurs, and phenolic compounds, showed various pharmacological properties and therapeutic effects.

**Objective:**

Anti-inflammatory, antioxidant, and immunomodulatory effects of *A. cepa* and its main constituents, along with the underlying molecular mechanisms are presented.

**Methods:**

Databases including, Web of Knowledge, Medline/PubMed, Scopus, and Google Scholar were checked for articles published between 1996 and the end of July 2020, using the key-words Allium cepa, quercetin, anti-inflammatory, antioxidant and immunomodulatory.

**Results:**

*A. cepa* and its constituents mainly quercetin showed anti-inflammatory effects mediated via reduction of total and differential WBC counts, inhibition of chemotaxis of polymorphonuclear leukocytes, COX, and LOX pathways and prevented formation of leukotrienes and thromboxanes, prostaglandin E2 (PGE2) as onVCAM-1, NF-κB, MARK,d STAT-1, JNK, p38 and osteoclastogenesis. *A. cepa* and its derivatives showed antioxidant effect by decreasing lipid peroxidation, NAD(P)H, MDA, NO, LPO and eNOS but enhancing antioxidants such as SOD, CAT, GSH, GPx, GSPO, TrxR, SDH, GST and GR activities and thiol level. Immunomodulatory effects of the plant and quercetin was also shown by reduction of Th2 cytokines, IL-4, IL-5, and IL-13 as well as IL-6, IL-8, IL-10, IL-1β and TNF-α and IgE levels, but increased CD4 cells, IFN-γ level and IFN-γ/IL4 ratio (Th1/Th2 balance).

**Conclusions:**

The effect of onion and its constituents on oxidative stress, inflammatory and immune system were shown indicating their therapeutic value in treatment of various diseases associated with oxidative stress, inflammation, and immune-dysregulation.

## Introduction

*Allium cepa* L. (Liliaceae) is commonly known as onion. The Liliaceae family includes over 250 genera and 3700 species (Nasri et al. 2012; Akash et al. 2014; Bisen and Emerald 2016). Onion, one of the oldest cultivated plants (Lanzotti 2006), originated from central Asia (Benkeblia 2004; Albishi et al. 2013) and is currently cultivated all over the world, particularly in zones with moderate climates (Nasri et al. 2012; Bisen and Emerald 2016). *A. cepa* is characterised by its colour (yellow, red, or white), and taste (sweet or non-sweet) (Benkeblia 2004; Albishi et al. 2013). It is consumed fresh in powder form, as an essential oil (Corea et al. 2005; Takahashi and Shibamoto 2008), and as a spice to enhance food flavour due to its odour and taste (Bouba et al. 2014).

*A. cepa* contains several constituents (Benmalek et al. 2013) and shows various pharmacological properties. The oldest usage of *A. cepa* was reported from ancient Egypt where it was used because of its antimicrobial, anti-inflammatory, and other healing properties (Dorsch et al. 1988). From ancient times, it has also been recognised as an effective treatment for stomach diseases, throat infection, and hepatitis (Akash et al. 2014). In Chinese medicine, *A. cepa* tea is used against fever, headache, cholera, dysentery, common cold, and arthritis (Corzo-Martínez et al. 2007). This plant was also used as an antifungal (Lanzotti 2006), anticancer, anti-inflammatory (Elberry et al. 2014), antioxidant, antispasmodic (Albishi et al. 2013; Benmalek et al. 2013), antimicrobial, antimutagenic (Shri and Bora 2008), antidiabetic (Ali et al. 2000; El-Aasr et al. 2010; Nasri et al. 2012), antiplatelet (Galmarini et al. 2001), and anti-asthmatic agent (Takahashi and Shibamoto 2008). Moreover, *A. cepa* showed antimicrobial, antithrombotic, antitumor, anti-hyperlipidaemic, anti-arthritic, anti-hyperglycemic anticarcinogenic properties (Upadhyay 2017).

Long-term consumption of *A. cepa* produced a preventive effect on the incidence of vascular and heart diseases, neurodegenerative disorders and cataract formation (Albishi et al. 2013). Other pharmacological properties shown for this plant include improvement of kidney function (Bisen and Emerald 2016), and anthelmintic, aphrodisiac, carminative, emmenagogue, and expectorant activities; also, it has beneficial effects on dysmenorrhoea, vertigo, fainting, migraine, wounds, scars, keloids, pain and swelling after bee or wasp stings, and is used for treatment of bruises, earache, jaundice, and pimples (Shri and Bora 2008).

*A. cepa* and its constituents have been extensively studied and several original and review articles were published on their pharmacological effects; the present article is an updated review of the studies that examined anti-inflammatory, antioxidants and immunomodulatory effects of *A. cepa* and its main constituents including flavonoid and phenolic compounds.

## Methods

The scientific databases, Web of Knowledge, Medline/PubMed, Scopus, and Google Scholar were searched to find studies on anti-inflammatory, antioxidants and immunomodulatory effects of *A. cepa* and its main constituents, published from 1996 until the end of July 2020. The following key words were used: ‘*Allium cepa’*, ‘onion’, ‘flavonoid’, ‘quercetin’, ‘organosulfur’, ‘saponin’, ‘phenolic compounds’, ‘anti-inflammatory’, ‘antioxidant’ and ‘immunomodulatory’.

### Chemical constituents

*A. cepa* contains water, carbohydrates, proteins, lectin, fats, vitamins, minerals, flavonoids, and organosulfur, and phenolic compounds (Benmalek et al. 2013). Interactions among the genotype, environment and agronomic practices influence the quality of onion constituents (Agnieszka et al. 2017).

Also, onion is a pool of free amino acids including aspartate (Asp), glutamate (Glu), asparagine (Asn), serine (Ser), glutamine (Gln), histidine (His), glycine (Gly), threonine (Thr), arginine (Arg), alanine (Ala), tyrosine (Tyr), methionine (Met), valine (Val), tryptophan (Trp), phenylalanine (Phe), isoleucine (Ile), leucine (Leu) and lysine (Lys) which supply the nitrogen content of onion and produce the characteristic taste called '*umami*' (Hansen 2001). Importantly, these amino acids in onion are potential fingerprints of geographical origin (Ianni et al. 2018). The amount of chemicals in onion varies based on the variety, geographical location and storage factors. Some varieties including yellow, red, and pink onions have high amounts of quercetin compared to white varieties. The highest level of flavonoids such as quercetin is observed in the dry skin and thus, peeling may significantly decrease these components and affect health benefits of onion. In addition, various cooking methods can affect flavonoid content of onion. Boiling significantly reduces flavonoids, microwaving has less effect on flavonoids content while frying results in the greatest loss (Hedges and Lister 2007).

There are several types of flavonoids present in the *A. cepa* in scales and disc of onion bulbs (Wiczkowski et al. 2008; Slimestad et al. 2007; Rodríguez Galdón et al. 2008; Ko et al. 2011; Shi et al. 2016; Rodrigues et al. 2017). More than 80% of the total content of flavonoids exists in the outer scales of *A. cepa* which is a result of exposure to sunlight. Free form of phenolic constituents is predominant in yellow and white onion skins, red onion flesh, sprouted flesh and green shoot (Albishi et al. 2013). Different types of onion significantly vary in terms of polyphenols. Yellow onion has the highest levels of flavonoids, 11-time higher than that of white onion. Red onion contains significant amounts of anthocyanin and has 10% flavonoid content (Yang et al. 2004; Slimestad et al. 2007).

Two groups of chemicals that are abundant in onions and have health benefits to humans, include flavonoids and alkenyl cysteine sulphoxides (Griffiths et al. 2002). About 31 unique proteins in the lower epidermis (LE) and upper epidermis (UE) of the scales of onion bulb were identified which involved in pigment synthesis, stress response, and cell division. Differences in chalcone-flavanone isomerase and flavone *O*-methyltransferase 1-like in LE of the onion scale, were shown to be responsible for red and yellow colours of onions. Also, differences in UDP-arabinopyranose mutase 1-like protein and β-1,3-glucanase in the LE, may be related to the differences in cell sizes in LE and UE of red and yellow onions (Wu et al. 2016). It was also shown that onions contain allylsulfides and flavonoids including quercetin which exert antioxidative activities and could reduce hepatocytes apoptosis; also, onion contains steroid saponins and sapogenins, and β-chlorogenin which is a characteristic steroid sapogenin. The other constituents of *A. cepa* are organosulfur compounds, including DATS, diallyl disulphide (DADS), ajoene, and sallylmercaptocysteine (SAMC), with cell cycle arrest effect in cancer cells (Upadhyay 2017).

Total polyphenols are 444.3–1591 mg/kg in garlic (*A. sativum* L.), chives (*A. schoenoprasum* L.), ramson (*A. ursinum* L.) and red, yellow and white onion (*A. cepa* L.) which decline in the order of chives > red onion > garlic > yellow onion > ramson > white onion (Lenková et al. 2016). Phytochemical analysis of *A. cepa* L. and *A. cornutum* using high-performance liquid chromatography (HPLC), showed that two quercetin conjugates, (1) and (2), account for about 80% of the total flavonol content in both onions (Fredotović et al. 2017).

The highest concentrations of quercetin, quercetin 3-β-d-glucoside, luteolin, and kaempferol in cv. ‘NHRDF Red’ (11,885.025 mg/kg), ‘Hissar-2′. (1432.875 mg/kg), ‘Pusa Riddhi’ (1669.925 mg/kg) and ‘Bhima Shakti’ (709.975 mg/kg), were found in dry skin of 15 Indian cultivars onion when their flavonoid concentration, total phenolic content (TPC) and total flavonoid content (TFC) were examined. In addition, cv. ‘NHRDF Red’ had the highest while cv ‘Bhima Shubhra’ had the lowest TPC and TFC content (Sagar et al. 2020). Detailed constituents of *A. cepa* are shown in Table 1.

**Table 1.** The major constituents of *Allium cepa*.

Major compounds	Minor compounds	Reference.
Water		Nile and Park (2014)
Proteins		Nile and Park (2014), Ashwini and Sathishkumar (2014)
Carbohydrates	Inulin, fructooligosaccharides, isorhamnetin-4-glucoside, galactose, glucose and mannose	Nile and Park (2014)
Vegetal hormone Lectin	Glycoquinine	Corzo-Martínez et al. (2007)
Steroids	Catechol, protocatechnic acid, thiocyanate and thiopropiono aldehyde	Ashwini and Sathishkumar (2014), Nile and Park (2014), Benmalek et al. (2013)
Phytoestrogens	Coumestrol, zearalenol, isoflavones and humulone	Ashwini and Sathishkumar (2014), Nile and Park (2014), Benmalek et al. (2013)
Vitamins	A, B complex, C and E	Ashwini and Sathishkumar (2014), Nile and Park (2014), Benmalek et al. (2013)
Minerals	Selenium, phosphorus, iron, calcium and chromium	Nile and Park (2014), Ashwini and Sathishkumar (2014)
Flavonoids	Quercetin, apigenin, rutin, myricetin, kaempferol, catechin, resveratrol, epigallocatechol-3-gallate, luteolin and genistein Quercetin aglycone, quercetin diglucoside, quercetin 4-glucoside and isorhamnetin monoglycoside or kaempferol monoglycoside	Ashwini and Sathishkumar (2014); Lanzotti (2006), Benmalek et al. (2013), Corzo-Martínez et al. (2007), Yamamoto and Yasuoka (2010), Nile and Park (2014), Rhodes and Price (1996), Wiczkowski et al. (2008), Shi et al. (2016), Rodríguez Galdón et al. (2008), Ko et al. (2011), Rodrigues et al. (2017), Slimestad et al. (2007)
Organosulfuric compounds	Thiosulphinates, cepaenes, cysteine, S-methyl cysteine sulfoxide, diallyl disulphide, allyl methyl sulphide, allyl propyl disulphide, gamma-L-glutamyl-trans-S-1-propenyl-L-cysteine sulfoxide, S-propenyl cysteine sulfoxide, S-alk(en)yl cysteine sulfoxides, S-allyl cysteine sulfoxide	Ashwini and Sathishkumar (2014), Lanzotti (2006), Nile and Park (2014), Benmalek et al. (2013)
Allicin	Diallyl disulphide, diallyl trisulphide and ajoene	Ashwini and Sathishkumarn (2014), Lanzotti (2006), Nile and Park (2014), Corzo-Martínez et al. (2007), Benmalek et al. (2013)
Phenolic compounds	Phenolics, phenolic acids, anthocyanins and hydroxycinnamic acid	Lanzotti (2006), Nile and Park (2014)
Lipophilic antioxidants	Dialkyl disulphides, aglycones, anthocyanin, saponins and fistulosin (octadecyl 3-hydroxyindole)	Ashwini and Sathishkumar (2014), Lanzotti (2006), Takahashi and Shibamoto (2008), Benmalek et al. (2013), Ernst and Feldheim (2000), Corzo-Martínez et al. (2007), Dorsch et al. (1990), Yamamoto and Yasuoka (2010), Nile and Park (2014), Griffiths et al. (2002), Augusti (1996), Rhodes and Price (1996), Khaki et al. (2009), Kuhnau (1976), Arjmandi et al. (1996)

### Anti-inflammatory effects of *A. cepa*

Inflammation is a defensive reaction of the body to eliminate damaging factors and re-establish domestic homeostasis. In the process of inflammation, blood flow increases in the damaged site due to the release of vasodilatory agents, capillary permeability enhances, and migration of white blood cells to the inflamed site is augmented; these lead to emergence of classic symptoms of inflammation namely, redness, warmth, swelling, pain, and in some cases, stiffness, and ultimately, damage to the affected area (O'Byrne and Dalgleish 2001). These changes are induced by cytokines and other inflammatory mediators (Dalgleish and O'Byrne 2002). Cytokines are classified into two major categories: pro-inflammatory and anti-inflammatory cytokines. Several cytokines including interleukin (IL)-1, tumour necrosis factor-alpha (TNF-α), IL-6 and IL-8, and chemokines such as granulocyte colony stimulating factor (G-CSF) and granulocyte-macrophage colony stimulating factor (GM-CSF) play a key role in acute inflammatory reactions (Rothwell et al. 1996). Several studies showed alleviative effects of onion and its active ingredients on inflammation and suggested that *Allium* plants are effective in treating inflammatory disorders at lower costs with limited side effects, in comparison to chemical drugs (Ali et al. 2000), as detailed below.

### Anti-inflammatory effects of the plant

Administration of aqueous extract of bulb of the red onion (EAC; 150 and 300 mg/kg) decreased eosinophil and lymphocyte counts in the blood and bronchoalveolar lavage fluid (BALF) in a Wistar rat model of asthma (Dawud et al. 2016). Anti-inflammatory and antibacterial effects of *A. cepa* were shown in previous studies and it was mentioned that this plant was extensively used for cure of catarrhal diseases, flu, angina, catarrh, cough, and prostatic hypertrophy (Kumar et al. 2010). The methanol extract *A. cepa* (50, 250 and 500 µg/mL) also showed a protective effect on neuroinflammation in lipopolysaccharide (LPS)-treated BV-2 microglial cells by reducing of pro-inflammatory cytokines TNF-α, IL-6, and IL-1-β (Jakaria et al. 2019).

It was observed that *A. cepa* skin extracts decreased cyclooxygenase-2 (COX-2) mRNA level in J774A.1 mouse macrophage cells treated with LPS (Albishi et al. 2013). In another study, intra-gastric intubation of juice of fresh onion bulbs at a daily dose of 1 mL sngiotensin converting enzyme (CE) for 14 days, inhibited COX and lipoxygenase (LOX) pathways and prevented formation of leukotrienes and thromboxanes in a male Sprague-Dawley rat model of doxorubicin-induced cardiotoxicity (Alpsoy et al. 2013)Aqueous extract of *A. cepa* also attenuated vascular inflammation by decreasing protein expression of vascular cell adhesion protein 1 (VCAM-1) (Alpsoy et al. 2013).

The bulb extract of *A. cepa* (35, 70, and 140 mg/kg/day for 21 days) significantly reduced total WBC and lung inflammatory cells such as neutrophil, eosinophil and monocyte counts, but led to a significant increase in lymphocytes counts in asthmatic Wistar rats (Ghorani et al. 2018) and the extract resulted in relaxation of tracheal smooth muscle (Memarzia et al. 2019). Also, the extract of onion reduced lung inflammatory cytokines such as IL-4, 5, and 13 and T helper 2 in BALF of asthmatic animals. These findings suggested therapeutic potential of *A. cepa* in the treatment of allergic disorders such as asthma (Oliveira, Campos et al. 2015). Chloroform extracts of yellow onion bulbs (0.3, 1, 3, 10 and 30 µM) inhibited M2 receptor in human monocyte-derived macrophages and suppressed the expression of CD163 which is involved in inflammation processes (El-Aasr et al. 2010). Methanol extract of the outer scales and edible parts of *A. cepa* bulb (100 and 200 mg/kg) showed protective effects on the infarct volume after cerebral ischaemia as reflected by a marked decrease in inflammation cascade in Swiss albino mice (Shri and Bora 2008). Onion peel hot water extract (0.1, 1, 10, 50, 100 µg/mL) administered to BALB/c mice, produced considerable anti-inflammatory activities as it could suppress the production of pro-inflammatory cytokines IL-6, TNF-α, and IL-1-β (Kang et al. 2015). It was shown that fresh onion juice (10 mg/kg) inhibited both acute and chronic pain and significantly decreased hind-paw thickness in male albino mice (Nasri et al. 2012). Steam distillate from freeze-dried *A. cepa* (methanol and water) attenuated 15-lipoxygenase type I activity which is an inflammatory mediator (Takahashi and Shibamoto 2008). Alcohol, chloroform and water extract of bulbs of *A. cepa* (300 mg/kg) showed wound healing activities in Wister albino rats which indicated the effect of the plant on initial phases of wound formation as an acute inflammatory process (Shenoy et al. 2009).

The anti-inflammatory properties of the plant against carrageenan-induced paw edoema in rats, were also investigated. Fresh onion juice was able to significantly decrease hind-paw thickness and demonstrated better results compared to the standard treatment, diclofenac (10 mg/kg). The anti-inflammatory effect of onion was attributed to it potential in preventing the formation of leukotrienes and thromboxanes via inhibition of COX and LOX pathways (Alpsoy et al. 2013).

### Anti-inflammatory effects of the constituents of *A. cepa*

It was reported that anti-inflammatory properties of *Allium* species are due to the presence of effective compounds such as tannin, flavonoids, anthocyanin, saponin, etc. (Aathira et al. 2020). *A. cepa* contains various flavonoids which can help in treatment of oxidative stress-mediated diseases, as well as inflammation, and thermal and mechanical hyperalgesia associated diseases (Vazhappilly et al. 2019).

Thiosulfinates and cepaenes found in *A. cepa*, can inhibit production of arachidonic acid as well as its downstream pro-inflammatory prostaglandins and leukotrienes (Wilson and Demmig-Adams 2007). Thiosulfinates and cepaenes (100 µM) exerted anti-inflammatory properties mediated through inhibition of chemotaxis of human polymorphonuclear leukocytes. It was also shown that cepaenes inhibit COX and LOX enzymes (Dorsch et al. 1990).

Quercetin, a well-known constituent of *A. cepa* showed several biological activities including reduction of swelling (inflammation), lung tightness and cholesterol and sugar levels in the blood (Hashemzaei et al. 2017; Aathira et al. 2020).

**Figure 1.**
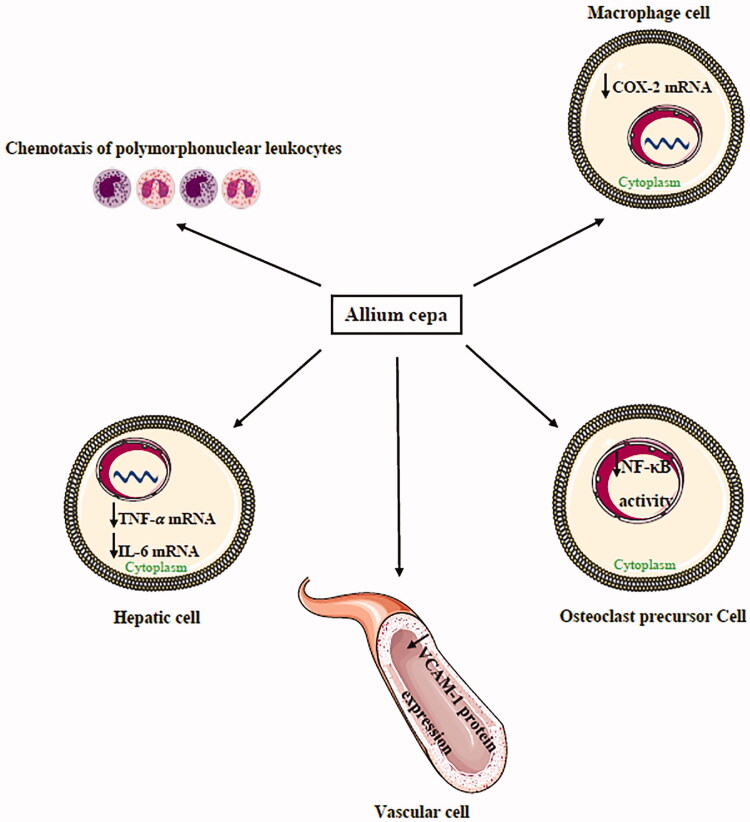
The anti-inflammatory effects of *Allium cepa* in different cells.: Decrease. COX-2: cyclooxygenase-2, IL-6: interleukin-6, NF-κB: nuclear factor kappa-light-chain-enhancer of activated B cells, TNF-α: tumour necrosis factor-alpha, VCAM-1: vascular cell adhesion protein 1.

Quercetin (0, 0.1, 1, 10, 25 and 50 µM) significantly suppressed nuclear factor kappa-light-chain-enhancer of activated B cells (NF-κB) induced by receptor activator of NF-κΒ ligand (RANKL) in MC3T3-E1 preosteoblastic cell line (Yamaguchi and Weitzmann 2011), (Figure 1). Quercetin also acts as a potent antioxidant and anti-inflammatory agent. This compound decreased the production of inflammatory cytokines such as IL-1α, IL-4, and TNF-α and inhibited the proliferation and activity of lymphocytes. In addition, quercetin reduced TNF-α/IL-10 and IL-8/IL-10 ratios in animal and human studies (Lanzotti 2006; Rivera et al. 2008; Boots et al. 2011). Umoh et al. (2019) showed that red onion is able to decrease inflammation by inhibition of NF-κB, MARK and STAT-1 possibly by its effective components such as quercetin.

Vazquez-Prieto et al. (2015) exanimated the effects of 6-week treatment with dietary catechin, quercetin, and a mixture of both, on tumour necrosis factor alpha (TNF-α) and adipose inflammation induced by high-fructose consumption, in Wistar rats. Catechin, quercetin, and their combination at the dose of 20 mg/kg/day, improved pro-inflammatory cytokines expression such as mitogen-activated protein kinase (MCP-1), resistin, and adipose tissue inflammation. In addition, catechin, quercetin, and their combination reduced the activation of the mitogen-activated kinases (MAPKs), JNK and p38. Also, catechin, quercetin, and their combination prevented downregulation of PPAR-γ.

Treatment with quercetin (0.1%) for 8 weeks, suppressed hepatic expression of TNF-α and IL-6 and decreased inflammation in high-fat diet/streptozotocin (STZ)-induced diabetic male Sprague-Dawley rats (Jung et al. 2011). It was also reported that quercetin was more effective in inhibiting inflammation processes compared to onion itself (Simin et al. 2013). *In vitro* studies showed that *A. cepa* extract and quercetin down-regulated NF-κB level and inhibited osteoclastogenesis in inflammatory conditions induced by LPS (Oliveira, Figueiredo et al. 2015). The anti-inflammatory activities of quercetin were attributed to its inhibitory effects on production of eicosanoids like thromboxane B2 (TXB2), prostaglandin E2 (PGE2) and 12 (*S*)-hydroxy-(5*Z*,8*Z*,10*E*,14*Z*)-eicosatetraenoic acid (12-HHT) which are inflammatory mediators derived from arachidonic acid (AA) (Lesjak et al. 2018). *A. cepa* (10, 100 and 1000 µM) and quercetin (3.5, 7.5 and 15 µM) also reduced the level of inflammatory cytokines such as IL-4, −5 and −13 in the BALF in a murine model of asthma (Oliveira, Campos et al. 2015). Some reports showed that allyl methyl disulphide (100 µM) from garlic had an anti-inflammatory effect on human colon cancer cell lines HT-29 and Caco-2 by increasing IL-8/IP-10 formation and suppressing IL-8 mRNA level in intestinal epithelial cells. Active butanolic fraction of ethanol extract of dried and grounded onion, quercetin and luteolin showed anti-inflammatory properties comparable to ibuprofen in rat peritoneal mast cells, and anti-edematogenic effect in the early phase of carrageenan-induced paw edoema (Zhang et al. 2015).

According to Simin et al. (2013), phenolic compounds in methanol extract of *Allium flavum* subsp. *flavum* or small yellow onion (71 and 81 µg/mL), including *p*-coumaric, caffeic, *p*-hydroxybenzoic, vanillic, protocatechuic and syringic acid, rutin, quercetin-3-*O*-glucoside and kaempferol-3-*O*-glucoside, expressed a high inhibitory potential for COX-1 and 12-lipoxygenase (12-LOX) activity. The methanol extract inhibited cell growth of cervix epithelioid carcinoma and colon adenocarcinoma cells. Phenolic compounds in soluble extracts of pearl onion skin at concentrations as low as 5 µg/mL, showed an antioxidant, anti-inflammatory and DNA scission inhibitory activity and inhibition of COX-2 expression and LDL cholesterol oxidation (Albishi et al. 2013).

Pre-treatment with the phenolic-rich extract of red onion peels (100 and 500 mg/kg) against oxidative stress induced by carbon tetrachloride (CCl_4_) free radicals in rat liver and kidney, ameliorated tissue levels of malondialdehyde (MDA) and significantly reduced the net carrageenan-induced edoema in the paw of rats (Ahmed et al. 2017).

The results of the above studies indicated the effect of *A. cepa* and its constituents such polyphenolics and flavonoids mainly quercetin, in inflammatory disorders of cardiovascular, gastrointestinal, neuronal respiratory and urogenital systems. The anti-inflammatory effects of the plant and its constituents were mediated via modulation of different inflammatory cells and mediators. Reduction of total WBC, neutrophils and eosinophil counts and inhibition of chemotaxis of human polymorphonuclear leukocytes, were reported in this context. The plant and its constituents showed inhibitory effects on COX and LOX pathways and prevented formation of leukotrienes and thromboxanes (such as TXB2), prostaglandin E2 (PGE2) and 12-HHT. Inhibitory effects of *A. cepa* and its constituents onVCAM-1, NF-κB, MARK,d STAT-1, JNK, p38 and osteoclastogenesis as well as downregulation of PPAR-γ were also shown. The results also indicated that the anti-inflammatory effects of the plant is due to its constituents mainly quercetin. The anti-inflammatory effects of *A. cepa* and its constituents are shown in Table 2.

**Table 2.** The anti-inflammatory effects of *Allium cepa* and its constituents.

Preparations	Doses	Model of study	Effects	Reference
Onion juice	10 mg/kg	Sprague-Dawley rat pain model	Inhibition of pain induced by inflammation	Nasri et al. (2012)
Methanolic Ext, outer scales and bulb	100 mg/kg and 200 mg/kg	Cerebral ischaemia model in Swiss albino mice	Reduction of infarct volume	Shri and Bora (2008)
methanol Ext, *A. flavum*	0.5-12 mg/mL	Human cell lines	Reduction of COX-1 and 12-LOX	Simin et al. (2013)
Allicin	5–20 mg/mL to >100 mM	Intestinal epithelial cells	Reduction of inhibition of TNF-α and IL-1β	Zhang et al. (2015)
*Allium cepa* Ext	1 mL/d for 14 days	Doxorubicin-induced cardiotoxicity Sprague-Dawley model	Inhibited COX and LOX pathways	Alpsoy et al. (2013)
*Allium cepa* Ext	35, 70 and 140 mg/kg/d, 21 days	Asthma model in Wistar rat	Reduced total WBC and lung inflammatory cells	Marefati et al. (2018)
Aqueous peel Ext quercetin	0.5 or 1% of OPE	STZ-induced diabetic Sprague-Dawley rats	Suppressed hepatic expressions of TNF-α and IL-6	Jung et al. (2011)
synthetic thiosulfinates, cepaene	0.1-100 microM	Human granulocytes chemotaxy	Inhibition of chemotaxis of leukocytes	Dorsch et al. (1990)
Quercetin	15 mg/d	Human study	Reduces the TNF-α/IL-10 ratio	Boots et al. (2011)
Quercetin	2 or 10 mg/kg/day, Gavage, 10 weeks	Zucker Rat	Reduction TNF-α	Rivera et al. (2008)
Quercetin	2.5 and 5 µM	LPS-Induced Osteoclastogenesis in murine macrophage cells	Decreased NF-κB, increased IL-3 and IL-4	Oliveira, Figueiredo et al. (2015)
Quercetin	30 mg/kg	Asthma murine model	Reduced IL4,5 and 13 in BALF	Oliveira, Campos et al. (2015)
Allyl methyl disulphide	0.5 and 1 mL/100 g bw/day	Human intestinal epithelial HT-29 cells	increase of IL-8/IP-10 suppression of the IL-8 mRNA	Zhang et al. (2015)
Phenolic compounds	0.125 and 0.5 mg/mL	J774A.1 mouse macrophage cells	Decreased COX-2 mRNA	Albishi et al. (2013)
Allicin	5–20 mg/mL to >100 mM	Intestinal epithelial HT-29 and Caco-2 cells	Reduction of TNF-α and IL-1β	Zhang et al. (2015)
Synthetic thiosulfinates, and cepaene	0.1-100 microM	Human granulocytes chemotaxy	Inhibition of chemotaxis of leukocytes	Dorsch et al. (1990)

Ref.: References, NF-κB: Nuclear Factor beta, COX: Cyclooxygenase, LOX: lipoxygenase, TNF-α: Tumour necrosis factoralpha, IL-1β: Interleukin 1 beta, bulf: bronco lung fluid, WBC: White blood cell, d: Day, Ext: Extract. COX: Cyclooxygenase, TNF-α: Tumour necrosis factoralpha, IL: Interleukin, Bulf: bronco lung fluid, d: Day.

### Antioxidant effects of *A. cepa*

Oxidative stress is characterised by over production of reactive oxygen species (ROS) and reactive nitrogen species (RNS) (Karimian et al. 2012). These free radicals, mainly nitric oxide, superoxide anion, hydroxyl radical and hydrogen peroxide, can cause oxidative damages to nucleic acids, proteins, and lipids. Thus, excess production of free radicals under pro-inflammatory conditions may initiate various diseases (Kim et al. 2012; Rezaee et al. 2014; Ghorani et al. 2018). Natural antioxidants are compounds that can delay or inhibit oxidative reactions by scavenging free radicals. The most important of these compounds are phenolic acids, polyphenols, flavonoids, alkaloids and terpenoids (Del Bano et al. 2006; Amidi et al. 2012; Kim IS et al. 2012). Therefore, suppression of oxidative stress could be achieved by using potential sources of natural antioxidants such as medicinal plants (Zarei et al. 2013; Parhiz et al. 2015; Boskabady et al. 2019; Hashemzaei et al. 2020). Essential oils derived from these plants, are rich sources of antioxidant components with different biological activities (Hasani-Ranjbar et al. 2009). *A. cepa* contains high levels of phenolic compounds mainly flavonoids, which have antioxidant properties besides other pharmacological effects such as antibiotic, antidiabetic, anti-atherogenic and anticancer activities (Helen et al. 2000; Liguori et al. 2017). Flavones, flavanones, flavonols, isoflavones, flavanonols, chalcones, and anthocyanins which are subclasses of flavonoids and flavonols, are the most abundant flavonoids in *A. cepa* (Liguori et al. 2017). Several studies reported the antioxidant activities of *A. cepa* and its constituents and introduced the plant as a potential source of natural antioxidants (Razavi and Kenari 2016; Roldán et al. 2008; Ola-Mudathir and Maduagwu 2014). Alterations in oxidant/antioxidant markers including lipid peroxidation (LPO), glutathione (GSH), superoxide dismutase (SOD), catalase (CAT), and MDA were observed by studies that investigated the effects of *A. cepa* and its constituents (Dadkhah et al. 2014).

### Antioxidant effects of the plant

Several studies examined the antioxidant capacity of onion essential oils and extracts using different methods including diphenyl-1-picrylhydrazyl (DPPH), β-carotene bleaching assays, azinobis (3-ethyl-benzothiazoline-6-sulfonic acid) (ABTS), oxygen radical absorbance capacity (ORAC) and Trolox equivalent antioxidant capacity (TEAC); findings confirmed remarkable antioxidant activity for the plant (Benkeblia 2005; Kim et al. 2010; Santas et al. 2010; Ye et al. 2013; Lisanti et al. 2016; Fredotović et al. 2017).

Razavi and Kenari (2016) reported the relationship between phenolic content and antioxidant activity of red onion peel extract using two methods of inhibition of free radical DPPH and β-carotene-linoleate bleaching assay. The results revealed that onion extract has a great antioxidant capacity and exhibited a significant relation between phenolic content and antioxidant activity of the plant. Furthermore, Lee et al. (2012) showed that Sprague-Dawley rats treated with red onion diet containing 5% red onion flesh for 4 weeks, exerted an elevation in the plasma SOD and glutathione peroxidase (GPx) activity.

Similarly, the antioxidant activity of onion peels extracts was assessed by DPPH radical scavenging activity. The results suggested that onion peel extracts have a remarkable antioxidant activity (Joung and Jung 2014).

In another study, the potency of methanol extract of *A. cepa* to scavenge free radicals, was examined by DPPH and ORAC methods which confirmed the extract’s phenolic compounds antioxidant effects (Fredotović et al. 2017). The radical scavenging and antioxidant activities of extracts of skin and edible part of *A. cepa*, were investigated by Škerget et al. (2009). Results showed a robust radical scavenging potential for the onion skin pure acetone extract, while the highest antioxidant activity was found for the onion skin extracted by 35 and 60% acetone and 60% ethanol. Also, a low antioxidant activity was seen for onion edible part in these experiments. Santas and colleagues (2010) reported the antioxidant properties of three different Spanish *A. cepa* cultivars including white skinned onion ‘Fuentes de Ebro’ white skinned onion ‘Calçot de Valls’ and yellow skinned onion ‘Grano de Oro’. Ethyl acetate sub-fraction contained the highest number of flavonoids and the TEAC was 74.86, 24.59 and 4.55 mol Trolox/g for ‘Grano de Oro,’ ‘Fuentes de Ebro’ and ‘Calçot de Valls,’ respectively. A potent antioxidant potential was also reported for the bulb methanol extracts of three *A. cepa* cultivars including ‘Pusa Red’ (red), ‘Pusa White Round’ (white) and ‘Arka Pitamber’ (yellow) (Santas et al. 2010) and a strong antioxidant activity was shown for red onion ‘N-530 from India (Singh et al. 2009). Benmalek and co-workers (2013) assessed the radical scavenging activity of *A. cepa* and showed an IC_50_ value of 2.91 × 10^−5 ^mg/mL for the outer layer of red onion.

Ren et al. (2017) measured the *in vitro* antioxidant activity of two onion varieties, Hyskin and Red Baron grown in a six-year field study. The effects of conventional, organic and mixed cultivation practices on phytochemical composition and antioxidant activity were also verified.

Gawlik-Dziki et al. (2013) measured the antioxidant potential of bread enriched with *A. cepa* skin. The food supplement was prepared by drying onions (‘Wolska’) in an oven at 50 °C followed by powdering the plant material using a laboratory mill. In these experiments, the flour used in the formula of control bread (wheat bread flour 600 g, type 750) was replaced with onion skin at 1, 2, 3, 4, and 5% levels. Bio-accessibility and bio-availability were determined *in vitro* using a human gastrointestinal tract model. Breads were then enriched with 80% methanol extract and antioxidant activity was measured in terms of antiradical activity, potential to suppress lipid peroxidation, metal chelating activity and ferric reducing power. The antioxidant activity of onion-supplemented bread was significantly higher than that seen for the control. Also, Helen et al. (2000) mentioned that 100 mg/kg onion oil given for 21 days is a potent antioxidant against oxidative injury caused by nicotine in Sprague-Dawley rats and its antioxidant activity was comparable to that of vitamin E.

*In vitro* antioxidant activity of methanol and aqueous extracts of *A. cepa* was compared using various methods such as DPPH and superoxide scavenging activity. The results showed that both extracts had antioxidant activity but this capacity was higher for the methanol extract of onion (Kaur et al. 2016). Various *in vitro* studies showed the presence of higher levels of antioxidants in oil and extracts of *A. cepa* using DPPH radical scavenging activity and other methods. *In vivo* evidence also confirmed potential antioxidant activity of the plant in different animal models (Votto et al. 2010; Cheng et al. 2013; Ye et al. 2013; Soto et al. 2015; Lenková et al. 2016; Shrestha et al. 2016; Ola-Mudathir et al. 2018).

In a Sprague-Dawley rat model, the antioxidant effect of *A. cepa* oil on nicotine-induced damages was compared to vitamin E. The results showed that 100 mg/kg/day onion oil given for 21 days led to significant increases in antioxidants (SOD, CAT and GSH) levels suggesting that *A. cepa* is an effective antioxidant against oxidative damage induced by nicotine (Helen et al. 2000).

Enhancement of antioxidant parameters such as SOD, CAT, thioredoxin reductase (TrxR), sorbitol dehydrogenase (SDH), and glutathione reductase (GR) and decrease of LPO were observed in the liver of mice infected with *Schistosoma mansoni* after treating with onion powder (2 g/100 g bw/day), (Mantawy et al. 2012). The protective effect of onion extract against doxorubicin-induced hepatotoxicity in rats, was also demonstrated. Doxorubicin, a chemotherapeutic agent, produces cardiotoxicity (Georgiadis et al. 2020) and hepatotoxicity via production of free oxygen radicals. However, significant reductions of MDA level, and increased levels of SOD, GSH and GPx were observed in Sprague-Dawley rats after treatment with 1 mL/day aqueous extract of onion, for 14 days (Mete et al. 2016). Similarly, aqueous extract of *A. cepa* (100 and 300 mg/kg/day) caused hepato-protective effects by improvement of antioxidant parameters such as SOD, CAT, GPx, GSH and MDA in alloxan-induced diabetic rabbits (Ogunmodede et al. 2011).

Cadmium, as a nephrotoxic agent, causes kidney damage through induction of oxidative stress. Preventive effect of *A. cepa* aqueous extract (1 mL for 8 weeks) against cadmium-induced renal dysfunction in a Wistar rats, was evaluated and the results showed significant improvements in plasma and tissue levels of SOD, CAT and MDA (Ige et al. 2009). Another study also showed that treatment of cadmium-intoxicated Wistar rats with aqueous extract of *A. cepa* (0.5 and 1 mL onion/100 g bw/day) for 7 days, led to a significant and dose-dependent restoration of renal oxidant (lipid peroxidation and glutathione-S transferase)/antioxidant (SOD, CAT and GSH) parameters (Suru 2008). The protective effects of methanol extract of *A. cepa* on cyanide-induced renal toxicity, were assessed in Wistar rats. Significant increases in antioxidant enzymes (SOD, CAT and GSH) and a significant reduction of MDA and LPO in the kidney, were observed in rats treated for 14 days with 600 mg/kg/day onion extract (Ola-Mudathir and Maduagwu 2014).

Administration of onion aqueous extract (0.5 and 1 mL onion/100 g bw/day) for 6 weeks, caused marked increases in hepatic and renal levels of GSH, GST, SOD and CAT, but significant reductions in MDA level in Wistar rats (Suru and Ugwu 2015).

In an *in vivo* study, the antioxidant effect of *A. cepa* juice (3 mL/day) on Wistar rat testis tissue and seminiferous tubules affected by *Escherichia coli*, was evaluated and the results showed significant increases in total antioxidant capacity after treatment of animals with *A. cepa* juice. Thus, this plant showed protective effects against *E. coli* infection-induced oxidative stress (Shahverdi et al. 2017). *A. cepa* aqueous extract (0.5 and 1 mL onion/100 g bw/day for 7 days) against cadmium-induced damage in prostate glands of Wistar rats produced significant improvements in oxidant/antioxidant status. These results suggested a chemoprotective capacity for this plant against biochemical alterations induced by cadmium in the prostate glands (Ola-Mudathir and Suru 2015). Protective effects of various doses (0.5 and 1.0 mL/100 g bw/day for one week) of onion aqueous extracts, were also indicated on sperm and testicular oxidative damage induced by cadmium in Wistar rats, which were mediated through reduction of LPO and MDA as well as improved antioxidant parameters (Ola-Mudathir et al. 2008).

The effects of *A. cepa* on the levels of oxidants and antioxidant markers in the BALF of ovalbumin-sensitised Wistar rats, were evaluated. Treatment with *A. cepa* juice (0.175, 0.35, or 0.7 mg/mL) significantly reduced oxidant markers such as nitrogen dioxide (NO_2_), nitrate (NO_3_^–^), and MDA but increased the levels of SOD and CAT in sensitised Wistar rats (Marefati et al. 2018).

Treatment of STZ-induced diabetic Wistar rats with aqueous extract of onion (0.4 g/mL/day) resulted in reduction of lipid hydroperoxide and lipoperoxide concentrations but did not alter GPx (Campos et al. 2003). Also, the level of free radicals was diminished in plasma and tissues of alloxan-induced diabetic rats after administration of onion extract (El-Demerdash et al. 2005) which was in agreement with previous studies (Baynes and Thorpe 1999; Kumari and Augusti 2002). Campos and co-workers (2003) examined the effects of the consumption of onion extract (40 g/100 mL for 30 days) in STZ-induced diabetic Wistar rats. It was shown that onion intake reduced SOD activity and prevented the increment of lipid hydroperoxide and lipoperoxide concentrations in treated diabetic rats. The antioxidant potential of the ethanol extract and fractions of aerial parts of A*. cepa* was examined by Baragob et al. (2015), in *in vitro* and *in vivo* studies. *In vitro* experiments used DPPH and NO radical scavenging methods, whereas the *in vivo* effects on antioxidant enzyme were investigated in the erythrocytes and pancreas of normal and STZ-induced diabetic albino rats. Before treatment, compared to diabetic groups, normal groups had higher levels of SOD, CAT, GSH but lower LPO level, while administration of *A. cepa* ethanol extract (200 mg/kg/day for 21 days) and its chloroform fraction, significantly augmented the levels of SOD, CAT and GSH and declined LPO level to near normal levels, in the diabetic groups.

Vazquez-Prieto et al. (2011) indicated anti-inflammatory and antioxidant effect of onion so that oral administration of onion extract (400 mg/kg/day for 8 weeks) in fructose-fed Wistar rats led to attenuation of lipid peroxidation and NAD(P)H oxidase activity and decreased heart endothelial nitric oxide synthase (eNOS) activity that are related to oxidative stress. Also, they found that the vascular inflammation was decreased through reduction of VCAM-1 expression.

In addition, the effects of processing technologies and storage conditions on antioxidant capacity of onions were investigated. Siddiq et al. (2013) showed that usage of mild-heat (50 and 60 °C) treatment for processing fresh-cut onions, did not affect the antioxidant activity (as assessed by ABTS and DPPH analysis) and the colour of fresh-cut onions.

The oxygen radical scavenging capacity of *A. cepa* was also reported (Kim et al. 2010). Pulp and skin of onion were extracted using distilled water and 95% ethyl alcohol. The highest ORAC value and total phenolic content were detected for the ethyl alcohol extract of onion skin. Lee et al. (2014) evaluated the antioxidant properties of four different extracts of *A. cepa* peels. Plant material was extracted by hot ethanol (60 °C), and hot water (80 °C) and by means of subcritical water extraction at 110 and 165 °C. The ethanol onion peel extract showed a better DPPH radical scavenging activity and a higher antioxidant activity compared to the other samples as determined by ferric thiocyanate assay.

Also, the antioxidant properties of the essential oil of *A. cepa* extracted by supercritical CO_2_ extraction were assayed by ABTS assay. The essential oil showed antioxidant (IC_50_ 0.67 mg/mL), using DPPH test (IC_50_ 0.63 mg/mL) and metal chelating assay (IC_50_ 0.51 mg/mL) (Ye et al. 2013).

In a clinical study, administration of 100 mL of onion juice daily for 8 weeks to subjects with mild hypercholesterolaemia, increased total antioxidant capacity and extended time of LDL oxidation (Lu et al. 2015) which was supported by other studies (Jain et al. 2018). Similarly, onion juice consumption (100 mL/day) for 8 weeks by healthy subjects, significantly improved total antioxidant capacity and levels of various antioxidants such as GSH and GR. Also, the levels of free radicals significantly reduced after treatment with the *A. cepa* (Law et al. 2016).

### Antioxidant effects of the constituents of *A. cepa*

Antioxidant effects of various constituents of *A. cepa* mainly quercetin, were shown in several studies. Treatment of hyperuricemic Wistar rats with 5 mg/kg quercetin for 14 days, led to a significant improvement in oxidative stress (Haidari et al. 2008). Pre-treatment of cortical neuronal cells derived from mouse embryos with quercetin (1-10 μM) for 30 min, protected cells from oxidative stress suggesting antioxidant effects of quercetin (Lee and Jung 2016).

It was also reported that oral administration of quercetin and piperine (100 mg/kg each) combination once a daily for 7 days, increased antioxidant and induced hepatoprotective effects against oxidative stress induced by paracetamol, in Wistar rats (Mehta et al. 2012). Quercetin oxidation metabolite, 2-(3,4-dihydroxybenzoyl)-2,4,6-trihydroxy-3(2H)-benzo-furanone (BZF), showed antioxidant effects at very low nanomolar concentrations (0.03 nM), in its pure form or as part of a plant extract. BZF protected human colonic adenocarcinoma cell line (Caco2 cells) against indomethacin-induced damage (Fuentes et al. 2020).

The antioxidant effects of active compounds extracted from medicinal herbs including monotropein from *Morinda officinalis* How (Rubiaceae), astragalin (kaempferol 3-*O*-glucoside) from *Cuscuta chinensis* Lamark (Convolvulaceae) and spiraeoside from the outer scales of *A. cepa* (200 mg/kg, oral) on varicocele-induced Sprague-Dawley rats significantly improved parameters of oxidative stress such as MDA, SOD and GPx (Karna et al. 2019).

Treatment of hepatitis induced by tetrachloromethane in Wistar rats, with dihydroquercetin (100 mg/kg, oral) for 4 days prior to the first administration of CCl_4_ and during the subsequent 14 days, indicated antioxidant effects of this agent (Teselkin et al. 2000).

Enhanced antioxidant capacity was shown for quercetin and quercetin + quercetin monoglycosides in the serum of Wistar rats fed with high-fat diets (Grzelak-Błaszczyk et al. 2018). Polysaccharide from *A. cepa* such as HBSS, CHSS, DASS and CASS, showed ABTS radical scavenging activity, DPPH radical scavenging activity, iron (Fe^2+^) chelating activity, and superoxide anion radical scavenging activity in a dose-dependent manner at concentrations of 0.5-2.0 mg/mL, and CHSS had the highest antioxidant action *in vitro* (Ma et al. 2018).

Free radical-scavenging activity of quercetin-3′-*O*-β-d-glucoside isolated from methanol extract of dried skin of *A. cepa*, was evaluated by ORAC assay and results showed that this component could be used as an antioxidant agent (Arung et al. 2011). Antioxidant properties, including OH radical scavenging effect of quercetin, isorhamnetin-3-glucoside, dipropyl disulphide and dipropyl sulphide extracted from methanol extract of *A. cepa*, were also demonstrated (Teshika et al. 2019).

Ouyang et al. (2018) showed DPPH radical-scavenging, FRAP radical scavenging, and OH radical scavenging activities of polyphenols from onion with IC_50_ values of 43.24, 560.61, and 12.97 μg/mL, respectively. In addition, these polyphenols significantly inhibited xanthine oxidase activity with an IC_50_ of 17.36 μg/mL. Insani et al. (2016) showed a positive correlation between total antioxidant activity and content of polyphenols particularly quercetin, suggesting that phenolic compounds have a major role in antioxidant properties of this plant. In another study, assessment of relationship between bioactive compounds content and antioxidant activities of 6 *Allium* vegetable species, revealed that chive onion had the highest antioxidant activity compared to others. A significant positive correlation between phenolic content and antioxidant activity was also shown (Beretta et al. 2017). Antioxidant effects of skin of 15 Indian cultivars onion showed the maximum antioxidant capacity for cv. ‘NHRDF Red’ whereas the least capacity was obtained for cv. ‘Bhima Shubhra’ (Sagar et al. 2020).

Together, the available literature indicated protective effects of *A. cepa* extract, fractions and its constituents especially quercetin, against several diseases associated with oxidative stress and lipid peroxidation in various body organs using different methods such as DPPH, ABTS, ORAC and TEAC. Treatment with *A. cepa* and its derivative mainly quercetin, decreased lipid peroxidation and NAD(P)H, MDA, NO, LPO and eNOS but enhanced antioxidant parameters such as total antioxidant capacity as well as SOD, CAT, GSH, GPx, GSPO, TrxR, SDH, GST and GR activities and thiol level. Also, free radical-scavenging activity such as OH radical scavenging effect was shown for the plant and its constituents. Therefore, onion and its component such as quercetin, may be used as an antioxidant agent in treatment of disorders associated with oxidative stress. In addition, the results showed that quercetin could be responsible for the antioxidant effects of the plant. Antioxidant effects of *A. cepa* and its constituents are summarised in Table 3.

**Table 3.** The antioxidant effects of *Allium cepa* and its constituents.

Preparations	Doses	Model of study	Effects	Reference
Essential oil	100 mg/kg/day, gavage	Nicotine-induced damages in Sprague–Dawley rats	Reduced LPO, increased SOD, CAT and GSH	Helen et al. (2000)
Onion powder	2 g/100 g body weigh/day, orally	Murine infected with *Schistosoma mansoni*	Reduced LPO, increased SOD, TrxR, SDH, GR	Mantawy et al. (2012)
Aqueous	1mL/day, orally	Doxorubicin-induced hepatotoxicity in Sprague-Dawley rats	Reduced MDA, increased SOD, GSH and GPx	Mete et al. (2016)
Aqueous	100 and 300 mg/kg/day, orally	Alloxan-induced diabetic rabbits	Increased SOD, CAT, GPx, GSH, reduced MDA content	Ogunmodede et al. (2011)
Aqueous	1 mL/day, orally	Cadmium-induced renal dysfunction	Improveed plasma and tissue levels of SOD, CAT and MDA	Ige et al. (2009)
Aqueous	0.5 and 1 mL/100 g bw/day, gavage	Cadmium-induced nephrotoxicity in Wistar rats	Reduced LPO and GST, increased SOD, CAT and GSH	Suru (2008)
Methanolic	600 mg/kg/day, orally	Cyanide-induced renal toxicity in Wistar rats	Reduced LPO and MDA, increased SOD, CAT and GSH	Ola-Mudathir and Maduagwu (2014)
Aqueous	0.5 and 1 mL/100 g bw/day, gavage	Endogenous hepatic and renal antioxidant status in Wistar rats	Decreased MDA, increased GSH, GST, SOD and CAT	Suru and Ugwu (2015)
Onion juice	3 mL/day, gavage	*Escherichia coli* induced testis and seminiferous tubules damage in Wistar rats	Enhanced total antioxidant capacity	Shahverdi et al. (2017)
Aqueous	0.5 and 1 mL/100 g bw/day, gavage	Cadmium-induced prostate glands damage in Wistar rats	Reduced GST, increased SOD, CAT and GSH	Suru and Ugwu (2015)
Aqueous	0.5 and 1 mL/100 g bw/day, gavage	Cadmium-induced sperm and testicular damage in Wistar rats	Reduced LPO, GST and MDA, increased SOD, CAT and GSH	Ola-Mudathir et al. (2008)
Onion juice	0.175, 0.35, or 0.7 mg/mL in drinking water	Wistar rat model of asthma	Decreased NO2, NO3^–^, MDA, elevated SOD and CAT	Marefati et al. (2018)
Aqueous	0.4 g/ mL/day, gavage	STZ-induced diabetic rats Wistar rats	Reduced lipid hydroperoxide and lipoperoxide	Campos et al. (2003)
Onion juice	100 mL/day, orally	Subjects with mild hypercholesterolaemia	Increased total antioxidant capacity and extended time of LDL oxidation	Jain et al. (2018), Lu et al. (2015)
Onion juice	100 mL/day, orally	Health subjects	Improved total antioxidant capacity, GSH and GR	Law et al. (2016)
Quercetin	5 mg/kg	Hyperuricemic Wistar rats	Improved oxidative stress	Haidari et al. (2008)
Quercetin	1–10 μM	Cortical neuronal cells	Protected cells from oxidative stress by inactivation of protein kinase C-ε	Lee and Jung (2016)
Quercetin	100 mg/kg, orally	Paracetamol induced oxidative stress in Wistar rats	Inhibited free radicals	Mehta et al. (2012)
Quercetin oxidation metabolite (BZF)	0.03 nanomolar	Indomethacin-induced damage in human Caco2 cells	Protected Caco2 cells against damage by antioxidant effect	Fuentes et al. (2020)
MAS	200 mg/kg, gavage	Varicocele-induced Sprague-Dawley rats	Improved parameters of oxidative stress such as MDA, SOD and GPx	Karna et al. (2019)
Dihydroquercetin	100 mg/kg, orally	CCl4- induced hepatitis in rat model	Hepatoprotective effect	Teselkin et al. (2000)
Polysaccharides	0.5-2.0 mg/mL	In vitro study	ABTS radical scavenging activity, DPPH radical scavenging activity, iron (Fe2+) chelating activity, and superoxide anion radical scavenging activity	Ma et al. (2018)

STZ: streptozocin; LPO: lipid peroxidation; SOD: superoxide dismutase; CAT: catalase; GSH: glutathione; TrxR: thioredoxin reductase; SDH: sorbitol dehydrogenase; GR: glutathione reductase; MDA: malondialdehyde; GPx: glutathione peroxidase; GST: glutathione S-transferase; NO_2_, nitrogen dioxide, NO3^–^: nitrate; BZF: 2-(3,4-dihydroxybenzoyl)-2,4,6-trihydroxy-3(2H)-benzo-furanone; Caco2: colonic adenocarcinoma cell line; MAS: MAS: monotropein, Astragalin (kaempferol 3-*O*-glucoside) and spiraeoside; ABTS: azinobis (3-ethyl-benzothiazolin-6-sulfonic acid); DPPH: diphenyl-1-picrylhydrazyl.

### Immunomodulatory effects of *A. cepa*

Immunomodulation is the process of moderating an immune response by administration of a chemical. The modulatory effects of several medicinal plants on cytokines and eventually, on the immune system were shown to be mediated through stimulation or suppression of various components of the immune system including the innate and adaptive immune responses (Spelman et al. 2006); immunomodulatory properties of *A. cepa* and its constituents have been widely evaluated by several studies (Spelman et al. 2006).

### Immunomodulatory effects of the plant

Several *in vitro* and *in vivo* studies reported the immunomodulatory effects of *A. cepa* in different diseases. In ovalbumin-sensitised Wistar rats, *A. cepa* aqueous extract (0.175, 0.35, and 0.7 mg/mL, oral) caused reductions in IL-4 and IgE levels, and increased the level of IFN-γ and IFN-γ/IL4 ratio (Th1/Th2 balance) indicating its stimulatory effect on Th_1_ but inhibitory effect on Th_2_ activity (Marefati et al. 2018).

An *in vitro* study on cultured spleen cells stimulated with pokeweed (PWM) from *Blomia tropicalis*-sensitised BALB/c mice, demonstrated that *A. cepa* methanol extract (10, 100 and 1000 μg/mL) inhibited the production of Th_2_ cytokines, IL-4, IL-5, and IL-13, and IgE (Oliveira, Campos et al. 2015). Also, oral administration of methanol extract of *A. cepa* (100 and 1000 mg/kg) attenuated the levels of IL-4, IL-5, IL-13, and IgE in BALF of a murine model of *Blomia tropicalis*-induced asthma (Oliveira, Campos et al. 2015). Zinc oxide nanoparticles (ZnO-NPs) synthesised from the extract of *A. cepa* (15 µg/mL) in UVB radiation-mediated inflammation in human epidermal keratinocytes (HaCaT cells), showed decreased levels such of IL-6, IL-10 and TNF-α (Wu et al. 2019).

Dietary administration of *A. cepa* (20 g/kg, oral) for 12 weeks, significantly increased weight gain, haematocrit, and total Ig in brown-marbled grouper fish compared to the control group (Apines-Amar et al. 2012). *A. cepa* scales ethanol extract (75, 150, and 300 mg/kg/day, oral) for 30 days, increased the tissue levels of IL-6, IL-8, and TNF-α and the expression of clusterin, while showing no effect on TGF-ΒR1 in Wistar rats with experimentally induced atypical prostatic hyperplasia (Elberry et al. 2014). In a similar study, ethanol extract of *A. cepa* (0.1, 1, 10, 50, and 100 μg/mL) in RAW264.7 cells, inhibited the secretion of IL-6, TNF-α, and IL-1β and the expression of COX-2, iNOS, NF-κB, and MAPKs in a dose-dependent manner (Ahn et al. 2015).

The effects of ethanol extract of *A. cepa* (100, 500, and 1000 μg/mL) on osteoclastogenesis under LPS-induced inflammatory conditions, were examined in RAW264.7 cells. Findings showed that *A. cepa* reduced the production of IL-6 and IL-1α, increased the production of IL-3 and IL-4, and down regulated NF-κB pathway (Oliveira, Figueiredo et al. 2015).

Stimulatory effects of ethanol extract of *A. cepa* (0.8–409.6 μg/mL) on lymphocyte response to a mitogen and IL-2 and IFN-γ gene expressions in white leghorn chickens, were evaluated (Hanieh et al. 2012). Oral administration of aqueous extract of *A. cepa* (250, 500 and 750 mg/kg) significantly increased CD4 cells in Wistar rats, indicating immunostimulatory potential of *A. cepa* (Mirabeau and Samson 2012).

Administration of *A. cepa* aqueous extract (0.1 mL/100 g bw, oral) for 7 weeks to female BALB/c mice with breast cancer, caused reductions in IL-4 and increases in IFN-γ level and IFN-γ/IL4 ratio (Th_1_/Th_2_ balance) indicating stimulatory effects of *A. cepa* on Th_1_ but inhibitory effects on Th_2_ activity (Karishchi and Bidaran 2018).

*A. cepa* (10 and 30 g/kg, oral) was administrated to white leghorn chickens immunised with Newcastle disease virus (NDV), sheep red blood cells (SRBC) and *Brucella abortus* (BA) which induced a dose-dependent increase in antibody titres higher than control, indicating stimulatory effect of *A. cepa* on humoral immune responses (Hanieh et al. 2010). Topical administration of two outer shells including the skin of *A. cepa* aqueous extract (20 and 40 µL,) five times a week for three consecutive weeks from day 21 to day 41 to BALB/c mice with allergic rhinitis, reduced allergic symptoms, eosinophil infiltration of nasal turbinate mucosa, and OVA-specific IgE levels. In addition, levels of IL-4, IL-5, IL-10, IL-13 and IFN-γ decreased in groups treated with onion extract (Seo et al. 2019).

Effects of ethanol extract of *A. cepa* (100 µg/mL) on LPS-induced inflammatory responses, were examined in RAW 264.7 cells and results showed reduced secretion of IL-6, TNF-α, and IL-1-β and NO production in a dose-dependent manner (Ahn et al. 2015). Methanol extract of *A. cepa* (50, 250, and 500 µg/mL) in LPS-induced BV-2 microglial cells (N27-A cells), reduced pro-inflammatory cytokines TNF-α, IL-6, and IL-1-β (Jakaria et al. 2019).

### Immunomodulatory effects of the constituents of *A. cepa*

Immunomodulatory effects of *A. cepa* constituents were also shown in various studies. Quercetin (3.5, 7.5, 15 μg/mL) inhibited production of Th2 cytokines, including IL-4, IL-5, IL-13, and IgE in cultured spleen cells stimulated with pokeweed (PWM) from *Blomia tropicalis*-sensitised BALB/c mice (Oliveira, Campos et al. 2015).

The effect of quercetin (1.25, 2.5 and 5 μM) on LPS-induced osteoclastogenesis in RAW264.7 cells, showed that quercetin reduced IL-6 and IL-1α, but increased IL-3 and IL-4 and down regulated NF-κB pathway (Oliveira, Figueiredo et al. 2015). The immunoprotective properties of *A. cepa* agglutinin (ACA) in normal and cyclophosphamide-induced immunosuppressed Wistar rats were demonstrated. ACA (1, 10, and 100 μg, intraperitoneal) increased TNF-α, IL-10, COX-2, IgG and IgA levels in serum and improved immune parameters such as cells of myeloid origin (RBC, WBC, and Hb), body weight, splenic index and thymic index in the spleen and thymus (Kumar and Venkatesh 2016).

The immunomodulatory activity of ACA (0.01, 0.1, 1 and 10 μg/well) was assessed in RAW264.7 cell and rat peritoneal macrophages. Results showed that ACA induced pro-inflammatory cytokines such as TNF-α and IL-12 and enhanced the proliferation of murine thymocytes and the expression of IFN-γ and IL-2; however, ACA caused no effect on proliferation of B cell-enriched rat splenocytes (Prasanna and Venkatesh 2015).

Onion fructo-oligosaccharides (FOS; 0.5, 5, 50 and 250 µg/mL) enhanced phagocytic activity in peritoneal exudates cells (PECs) in Wistar rats and cell proliferation or mitogenicity of splenocytes and thymocytes in BALB/c mice (Kumar et al. 2015). Lectin as an effective constituent of *A. cepa* also showed remarkable immunoprotective effects and elevated the levels of proinflammatory COX-2 and nitric oxide and expression of immunoregulatory cytokines TNF-α, IL-2, IL-12 and IFN-γ (Prasanna and Venkatesh 2015; Kumar and Venkatesh 2016).

Total phenol content (TLC) of Toscana (red onion) bulb extract were tested on immunological cells such as T helper cells (CD4+ cells), cytotoxic T lymphocytes (CD8+ cells), T regulatory cells (CD25high CD4+ cells), and natural killer cells/monocytes (CD16+ cells). The results showed that TLC increased the frequency of antitumor/anti-infection NK CD16+ immune cells (Lisanti et al. 2016). In addition, polyphenols extracted from lyophilised *A. cepa* (100 mg/mL) inhibited cancer cell growth by induction of caspase-dependent apoptosis through suppressing caspase 8 and 9 and up-regulation of TNF-related apoptosis-inducing ligand (TRAIL) receptor DR5 and down-regulation of the cellular inhibitor of apoptosis 1 (cIAP-1). Also, polyphenols inhibited phosphatidylinositol 3-kinase (PI3K)/Akt signalling pathway in human leukemic cells and U937 cells (Han et al. 2013).

The reviewed *in vitro* and *in vivo* studies showed the modulatory effects of *A. cepa* and its constituents on the immune system in various immune dysregulatory disorders. The plant and its components mainly quercetin, reduced Th2 cytokines, IL-4, IL-5, and IL-13 as well as IL-6, IL-8, IL-10, IL-1β and TNF-α and IgE levels, but increased CD4 cells, IFN-γ level and IFN-γ/IL4 ratio (Th1/Th2 balance), indicating their stimulatory effect on Th1 but inhibitory effect on Th2 activity in inflammatory disorders such as asthma and breast cancer. However, under inflammatory conditions such as osteoclastogenesis induced by LPS in RAW264.7 cells, *A. cepa* and quercetin reduced IL-6 and IL-1α production, but increased IL-3 and IL-4 levels. In animal models of allergic rhinitis, the plant reduced allergic symptoms, eosinophil infiltration of nasal turbinate mucosa, and OVA-specific IgE as well as IL-4, IL-5, IL-10, IL-13 and IFN-γ levels. Therefore, the varying types of immunomodulatory effects of *A. cepa* and its constituents were observed in different immune dysregulaton disorders. The above studies showed that *A. cepa* and its constituents especially quercetin, are potential immunomodulatory therapeutic candidates for treatment of disorders with immune dysregulation. Table 4 illustrates immunomodulatory effects of *A. cepa* and its constituents. Based on the described studies, quercetin contributes to immunomodulatory effect of the plant.

**Table 4.** The immunomodulatory effects of *Allium cepa* and its constituents.

Preparations	Dose	Study models	Effects	Ref.
*A. fistulosom*	2.5, 5 and 10 mg/400 mL /mouse 125–1000 µg/mL	Murine macrophage cell line RAW264.7	Increased TNF-α, IL-12, IFN-γ production, phagocytosis, NK cell activities, increased TNF-α, IL-12, and MCP-1 production	Ueda et al. (2013)
AE	0.175, 0.35, and 0.7 mg/mL, orally	OVA-sensitised Wistar rat	Decreased IL-4 and IgE, increased IFN-γ and IFN-γ/IL-4 ratio	Marefati et al. (2018)
AE	20 and 40 μL, orally	OVA-sensitised BALB/c mice	Reduced the levels of IL-4, IL-5, IL-10, IL-13 and IFN-γ	Seo et al. (2019)
	100 µg/ mL	Murine macrophage cell line RAW264.7		Ahn et al. (2015)
EE		LPS-induced inflammatory markers in BV-2 microglial cells	Reduced NO production and IL-6, TNF-α, and IL-1β secretion	
ME	50, 250, and 500 µg/mL	Testosterone induced atypical prostatic hyperplasia in Wistar rats	Reduced TNF-α, IL-6, and IL-1β	Jakaria et al. (2019)
*Allium cepa*	75, 150, and 300 mg/kg		Reduced tissue expressions of IL-6, IL-8, TNF-α and, IGF-1	Elberry et al. (2014)
ME	10, 100 and 1000 μg/ml	PWM-stimulated splenocytes from Bt-sensitised BALB/c mice	Reduced IL-4, IL-5, IL-13, and IgE	Oliveira, Figueiredo et al. (2015)
ME	100 and 1000 mg/kg, orally	Bt-sensitised BALB/c mice	Reduced the levels of IL-4, IL-5, IL-13, and IgE in BALF	Oliveira, Campos et al. (2015)
*Allium cepa*	20 g/kg, orally	Brown-marbled grouper fish	Increased weight gain, haematocrit, and total Ig	Apines-Amar et al. (2012)
EE	75, 150, and 300 mg/kg/day, orally	Testosterone-induced APH in Wistar rat	Increased IL-6, IL-8, and TNF-α and the expression of clusterin	Elberry et al. (2014)
EE	0.1, 1, 10, 50, and 100 μg/mL	Murine macrophage cell line RAW264.7	Inhibited IL-6, TNF-a, and IL-1ß secretion and COX-2, iNOS, NF-κB, and MAPKs expression	Ahn et al. (2015)
ME	100, 500, and 1000µg/mL	Murine macrophage cell line RAW264.7	Reduced IL-6 and IL-1α production, increased IL-3 and IL-4 production, Downregulated NF-κB pathway	Oliveira, Campos et al. (2015)
EE	0.8–409.6 μg/mL	Lymphocyte isolated white leghorn chickens	Lymphocyte response to a mitogen Stimulation and IL-2 and IFN-γ gene expressions	Hanieh et al. (2012)
AE	250, 500 and 750 mg/kg, orally	Wistar rat	Increased the CD4 cells	Mirabeau and Samson (2012)
AE	0.1 mL /100 gBW, orally	Breast cancer-induced by cell line 4T1	Reduced IL-4, increased IFN-γ	Karishchi and Bidaran (2018)
*Allium cepa*	10 and 30 g/kg, orally	Chickens immunised with NDV, SRBC and BA vaccines	Improvement of efficacy of vaccines	Hanieh et al. (2010)
Quercetin	3.5, 7.5, 15 μg/mL	PWM-stimulated splenocytes from Bt-sensitised BALB/c mice	Reduced IL-4, IL-5, IL-13, and IgE	Oliveira, Figueiredo et al. (2015)
Quercetin	30 mg/kg, orally	Bt-sensitised BALB/c mice	Reduced the levels of IL-4, IL-5, IL-13, and IgE in BALF	Oliveira, Figueiredo et al. (2015)
Quercetin	1.25, 2.5 and 5 µM	Murine macrophage cell line RAW264.7	Reduced IL-6 and IL-1α production, increased IL-3 and IL-4 production, Downregulated NF-κB pathway	Oliveira, Campos et al. (2015)
ACA	1, 10, and 100 μg, i.p.	CP- immunosuppressed Wistar rats	Increased serum TNF–α, IL-10, COX–2, IgG and IgA, improved immune parameters in spleen and thymus	Kumar and Venkatesh (2016)
ACA	0.01, 0.1, 1 and 10 μg/well	Murine macrophage cell line RAW264.7	Stimulated TNF-α and IL-12 production, enhanced murine thymocytes proliferation and IFN-γ and IL-2 expression	Prasanna and Venkatesh (2015)
FOS	0.5, 5, 50 and 250 µg/mL	Splenocytes and thymocytes	Increased PECs phagocytic activity, cell proliferation or mitogenicity	Kumar et al. (2015)

Ref.: References; AE: aqueous extract; ME: methanolic extract; EE: ethanoic extract; ME: methanolic extract; CE: chloroformic extract; PWM: pokeweed; Bt: *Blomia tropicalis*; CP: cyclophosphamide; APH: atypical prostatic hyperplasia; FOS: onion fructo-oligosaccharides; PECs: peritoneal exudates cells; ACA: *Allium cepa* agglutinin; SRBC: sheep red blood cells; NDV: newcastle disease virus; SRBC: sheep red blood cells; BA: brucella abortus.

**Figure 2.**
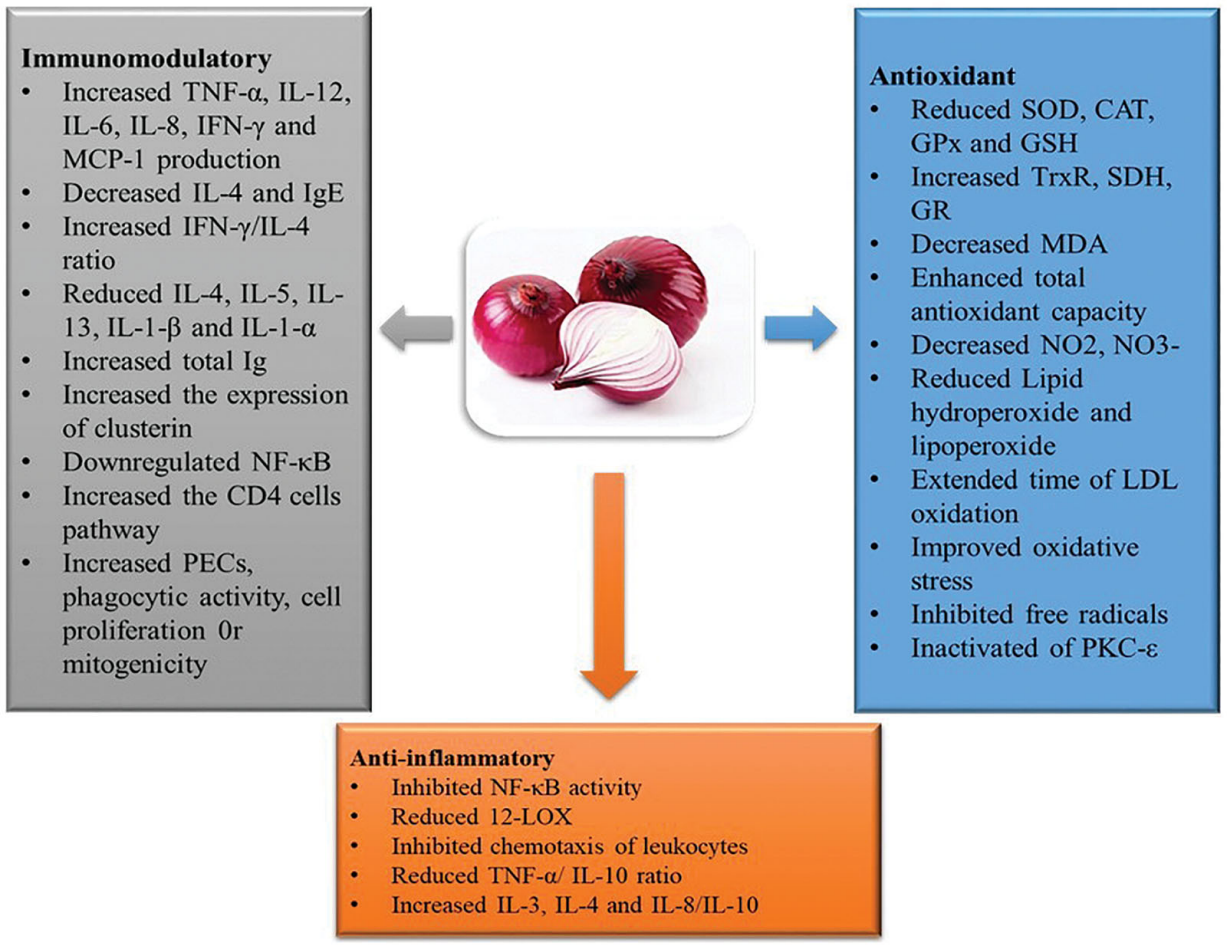
A summary of the possible mechanisms of onions.

This review highlighted anti-inflammatory, antioxidant, and immunomodulatory effects of *A. cepa* and its major constituents, mediated via mechanisms that are depicted in Figure 2.

## Conclusions

This review discussed the effects of *A. cepa* (onion) and its constituents on inflammation, oxidative stress and immune disorders as shown by *in vitro* and *in vivo* studies.

*A. cepa* and its components showed antidotal effects in different pathological conditions induced by various chemicals/toxins. Together, the plant and its components showed anti-inflammatory and immunomodulatory effects mediated by modulation of innate (neutrophils, macrophage, and NK cells) and acquired immunity components (inflammatory and anti-inflammatory cytokines, B cells, and Th_1_/Th_2_ balance).

Antioxidant effects of *A. cepa* and its constituents were mediated through stabilisation of cellular membranes, ROS scavenging, and decrement of unsaturated membrane lipids peroxidation. Therefore, *A. cepa* and its constituents could be of therapeutic value in disorders such as aging, anti-inflammatory, and wound healing processes where radical scavenging activity can be of therapeutic value.

Although the exact molecular mechanisms underlying such effects are not fully understood yet, most of pharmacological activities of *A. cepa* are related to the presence of bioactive compounds such as quercetin. Further clinical studies are needed to evaluate the effect of the plant and its constituents on conditions induced by inflammation, oxidative stress and immune-dysregulation. In addition, scientific information on toxicity or safety of onion is lacking and requires further studies.
